# Membranous NOX5-derived ROS oxidizes and activates local Src to promote malignancy of tumor cells

**DOI:** 10.1038/s41392-020-0193-z

**Published:** 2020-08-14

**Authors:** Jie Chen, Yan Wang, Weimin Zhang, Di Zhao, Lingyuan Zhang, Jiawen Fan, Jinting Li, Qimin Zhan

**Affiliations:** Key laboratory of Carcinogenesis and Translational Research (Ministry of Education/Beijing), Laboratory of Molecular Oncology, Peking University Cancer Hospital & Institute, 100142 Beijing, China

**Keywords:** Gastrointestinal cancer, Gastrointestinal cancer

## Abstract

Reactive oxygen species (ROS) localized at the precise subcellular compartments are essential for regulating the activity of signaling proteins. Furthermore, ROS are master regulators of tumor malignant progression that respond to a diverse set of environmental stress, especially hypoxia. NADPH oxidases (NOXs) appear to be activated within discrete subcellular compartments to facilitate local ROS production. However, the subcellular function of NOXs in hypoxic tumor is still unclear. In this study, we demonstrated that NOX5 was greatly upregulated in clinical esophageal squamous cell carcinoma (ESCC) tumors, ESCC cell lines or primary ESCC cells, and elevated NOX5 was correlated to malignancy of ESCC tumors and poor prognosis. NOX5 induced the malignant progression of ESCC by activating Src, especially under hypoxic condition. Mechanistically, we showed that hypoxia promoted the interaction between NOX5 and Pyk2 on cell membrane via facilitating Ca^2+^-mediated Pyk2 Tyr^402^ site phosphorylation. Subsequently, Pyk2 acted as a scaffold for c-Abl phosphorylating the catalytic domain of NOX5 Tyr^476/478^ sites, which in turn upregulated hydrogen peroxide (H_2_O_2_) inside the Pyk2/NOX5 complex to oxidize and activate local Src. These findings provide insights into the biological significance of NOX5 in the development of ESCC.

## Introduction

Reactive oxygen species (ROS) are diffusible and short-lived signaling molecules, which induce various biological events. ROS at the specific subcellular compartment are critical for regulating redox-dependent signaling pathways under environmental stresses. Among these environmental stresses, hypoxia is an important prognostic and predictive factor owing to its multiple contributions to proliferation, invasiveness, metastasis, angiogenesis, resistance to cell death and altered metabolism in the process of tumor progression.^1,2^ Hypoxia activates many of the known oncogenic signaling proteins through stimulating the production of intracellular ROS to induce tumor malignant progression.^3,4^ Gastrointestinal epithelial cells are sensitive to environment stress to produce ROS, which gradually induce the transformation of these cells and lead to gastrointestinal tumors.^5–7^ However, the exact mechanisms by which gastrointestinal tumor cells, especially ESCC, sense hypoxia, integrate and activate components of oncogenic signaling pathways via local ROS are still unclear.

NADPH oxidases (nicotinamide adenine dinucleotide phosphate oxidase, NOXs) are a family of enzymes with the primary function to generate ROS. They consist of seven members, represented by different catalytic subunits: NADPH oxidase 1 (NOX1) to NOX5, dual oxidase 1 (DUOX1), and DUOX2. NOXs use different regulatory subunits to produce ROS.^8,9^ NOXs in specific cellular microdomains, such as lamellipodia, membrane ruffles or endosomes, can interact with signaling platforms to provide a redox-dependent effect and resultantly achieve localized ROS production. For example, tyrosine kinase substrates (TKS) proteins, TKS4 and TKS5, interact with NoxA1 proline rich region (PRR) to enhance NoxA1-NOX1 binding and subsequently cause NOX1 localization to invapodia and increased ROS production.^10^ Pyk2/Grb2 recruits NOX4 to the scaffold protein SHPS-1, and NOX4 locates to cell membrane to activate Src in human vascular smooth muscle cells (VSMCs) treated with IGF-1.^11^ Increasing studies have shown that NOXs promote cancer progression via stimulating oncogenic signalings.^12–17^ Nevertheless, the cofactors that facilitate NOXs function in specific subcellular compartments to activate signaling pathways and promote cancer progression remain a huge puzzle.

In this study, we intended to evaluate the expression of all members of the NOXs family in ESCC and establish the correlation between NOX5 expression and tumor malignancy. Importantly, we studied the role of NOX5 in regulating the malignant progression of ESCC cells and explored the underlying mechanisms.

## Results

### NOX5 is frequently upregulated in human ESCC cells

**Fig. 1:**
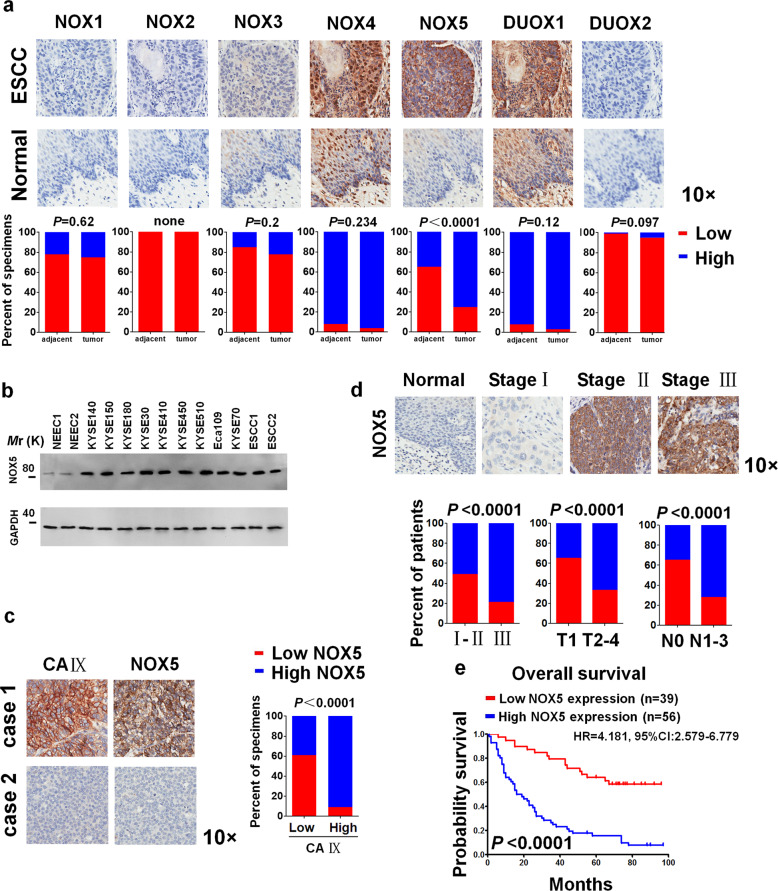
NOX5 positively correlates with the progression of ESCC. **a** Immunohistochemical staining evaluated the expression of NOX1-5, or DUOX1, 2 in 92 pairs of ESCC, and their respective adjacent noncancerous tissues (cohort I). Representative results of immunohistochemical staining for NOX1-5, or DUOX1, 2 in the same set of consecutive tumor tissue and their respective adjacent noncancerous tissue slices. Magnification, ×10 as indicated. The chi-square test was employed to analyze correlations between tumors and their respective adjacent normal tissues. **b** Immunoblotting analysis of NOX5 in primary normal human esophageal epithelial cells (NEECs) and cultured ESCC cell lines or primary ESCC cells. GAPDH was used as a loading control. **c** Representative results of immunohistochemical staining for CA IX and NOX5 in the same set of consecutive tumor tissue slices (cohort I). High NOX5 levels significantly correlates with high level CA IX, examined using the chi-square test. **d** The chi-square test was employed to analyze correlations between different clinical parameters, including tumor stage, tumor status and lymph node status, without adjustments (ESCC: *n* = 95 biologically independent samples; cohort II). **e** Kaplan–Meier curves of ESCC patients with low versus high expression of NOX5 (*n* = 95; HR = 4.181, 95% CI: 2.579–6.779, *P* < 0.0001, log-rank test). Significant differences were compared using the log-rank test (two-sided) without adjustments

To assess the expression of NOXs family in patients with ESCC, we conducted the analysis of the protein expression of NOX1-5, DUOX1, 2 in 92 pairs of ESCC and adjacent normal tissues (cohort I) using immunohistochemistry (IHC) assay. The protein levels of NOX5 were significantly upregulated in these ESCC samples in contrast to their adjacent normal tissues (Fig. 1a, and Supplementary Table 1). Negative control staining of tumor slide was shown in Supplementary Fig. 1. Immunoblotting analysis clearly showed that the protein expression of NOX5 was significantly higher in primary ESCC cells or ESCC cell lines, compared with normal epithelial esophageal cells (NEECs) (Fig. 1b). Importantly, the staining of carbonic anhydrase IX (CA IX), a marker for tumor hypoxia^18,19^ was positively correlated with NOX5 in ESCC samples (cohort I; Fig. 1c).

Then, we explored the clinical relevance of NOX5 expression in 95 cases of patients with ESCC (cohort II). Strong expression of NOX5 was positively correlated with advanced-stage, higher grade tumor status and higher grade lymph node status (Fig. 1d, and Supplementary Table 2). Moreover, the patients with higher expression of NOX5 showed a shorter overall survival time determined by the Kaplan–Meier and log-rank tests (Fig. 1e).

### NOX5 activates Src in ESCC cells

**Fig. 2:**
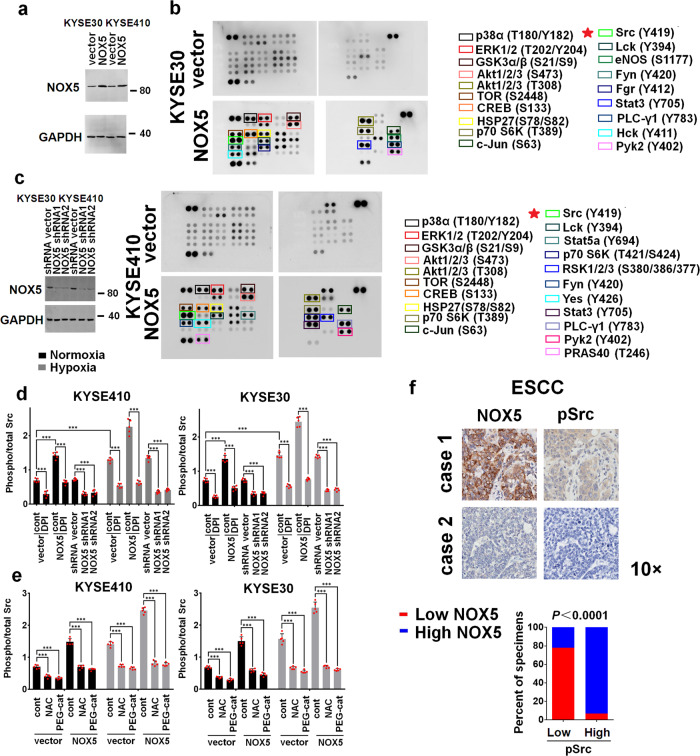
NOX5 positively regulates Src activity. **a** Stable overexpression of NOX5 in the indicated ESCC cell lines tested by immunoblotting. GAPDH was used as a loading control. **b** Total protein lysates from the vector control or NOX5-overexpressing KYSE30 (upper panel) or KYSE410 (lower panel) cells were analyzed using antibody array against 43 kinase phosphorylation sites. **c** Silencing NOX5 in two specific short hairpin (sh) RNA-transduced stable ESCC cell lines examined by immunoblotting. GAPDH was used as a loading control. **d** vector control or NOX5-overexpressing KYSE30 (right panel) or KYSE410 (left panel) cells were pretreated with 10 μM NADPH oxidase inhibitor-DPI or control solvent for 90 min, respectively, or shRNA vector or NOX5 shRNA-1, shRNA-2, were cultured under normoxic and hypoxic conditions for 24 h. The Src activity was assayed by Src activation quantitative ELISA assay. **e** vector control or NOX5-overexpressing KYSE30 or KYSE410 cells were treated with ROS scavenger NAC (2 mM, pretreated with 90 min) or H_2_O_2_ scavenger-PEG-catalase (400 units/ml) or control solvent, respectively, were cultured under normoxic and hypoxic conditions for 24 h. Src activity was assessed using quantitative ELISA assay. ***P < 0.001; two-tailed unpaired Student’s *t*-test. Error bars represent mean ± SD of five independent experiments. **f** NOX5 expression was associated with pSrc (Tyr^419^), expression in 92 primary human ESCC specimens (cohort I). Two representative specimens with low and high levels of NOX5 were shown. Magnification, ×10 as indicated. Percentages of specimens showing low or high NOX5 expression relative to the level of pSrc. Statistical differences were evaluated using the chi-square test

We next screened NOX5-regulated pathway kinases in ESCC cells using phospho-kinase array. As shown in Fig. 2a and b, several kinases were activated by NOX5 in ESCC cells, especially Src. Correspondingly, overexpression or knockdown of NOX5 effectively upregulated or downregulated the Src activity under both normoxic and hypoxic conditions, evaluated by quantitative ELISA assays (Figs. 2c–e). Figure 2d–e revealed that NADPH oxidase inhibitor diphenyleneiodonium (DPI, 10 μM, pretreated with 90 min), ROS scavenger *N*-Acetyl-L-cysteine (NAC, 2 mM, pretreated with 90 min) or hydrogen peroxide (H_2_O_2_) scavenger-PEG-catalase (400 units/ml) effectively inhibited NOX5-mediated Src activation, under either normoxic or hypoxic condition. These results together indicate that NOX5 activates Src in ESCC cells. We also examined whether the NOX5/Src axis was clinically relevant. As shown in Fig. 2f, NOX5 was strongly correlated with pSrc (*P* < 0.0001) in 92 ESCC specimens.

### Hypoxia induces the interaction Pyk2/NOX5 on cell membrane in ESCC cells

**Fig. 3:**
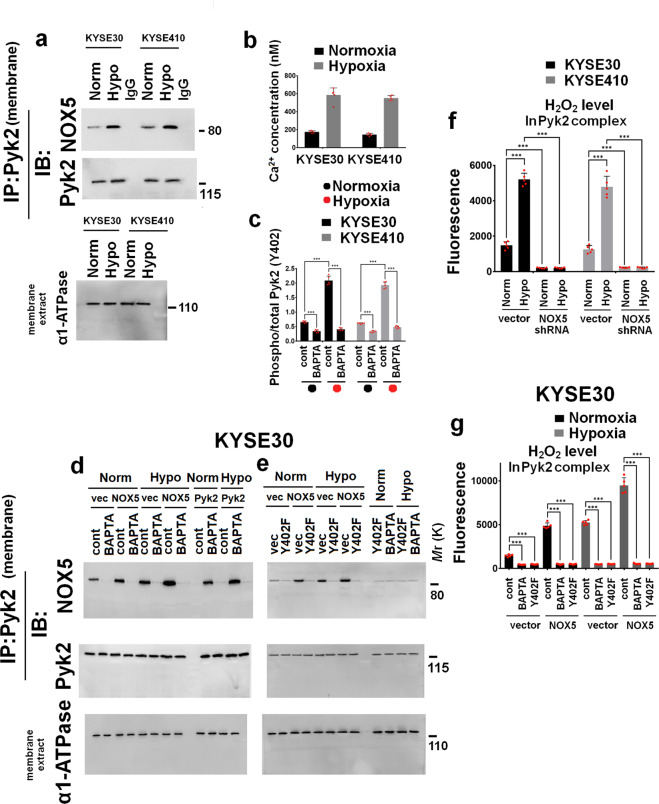
Hypoxia induces cell membranous interaction between Pyk2 and NOX5. **a** KYSE30 and KYSE410 cells were cultured under normoxic or hypoxic condition for 1 h. Cell membrane lysates were immunoprecipitated with Pyk2 antibody. Immunocomplexes were then immunoblotted using NOX5 and Pyk2 antibodies. The efficacy of membrane protein extraction was examined using immunoblotting to detect the expression of α1-ATPase (membrane biomarker) in cell membrane lysis. **b** KYSE30 and KYSE410 cells were cultured under normoxic or hypoxic condition for 1 h. Intracellular Ca^2+^ level was evaluated using Calcium detection assay kit. **c** KYSE30 and KYSE410 cells were pretreated with 10 μM Ca^2+^ chelator-BAPTA-AM for 30 min and then exposed to normoxic or hypoxic condition for 1 h. The Pyk2 (Tyr^402^) phosphorylation was assayed by Pyk2 activation ELISA assay. **d**, **e** KYSE30 control, NOX5, or Pyk2-ovexexpressing cells were pretreated with 10 μM Ca^2+^ chelator-BAPTA-AM for 30 min or control solvent (**d**), or transfected with control vector or Pyk2 Y402F plasmid (**e**), were cultured normoxic or hypoxic condition for 1 h. Cell membrane lysates were immunoprecipitated with Pyk2 antibody. Immunocomplexes were then immunoblotted using NOX5 and Pyk2 antibodies. The efficacy of membrane protein extraction was evaluated using immunoblotting to assess the expression of α1-ATPase (membrane biomarker) in cell membrane lysis. **f** The KYSE30 and KYSE410 control shRNA or NOX5 shRNA cells were exposed to hypoxia for 1 h. Cell membrane lysates were immunoprecipitated with Pyk2 antibody. Then, the Pyk2 complex-produced H_2_O_2_ was examined using an Amplex red hydrogen peroxide assay kit. **g** KYSE30 control or NOX5-overexpressing cells pretreated with or without 10 μM Ca^2+^ chelator-BAPTA-AM, or transfected with control vector or Pyk2 Y402F plasmid, were exposed to normoxic or hypoxic condition for 1 h. Cell membrane lysates were immunoprecipitated with Pyk2 antibody. Then, the Pyk2 complex-produced H_2_O_2_ was assayed using an Amplex red hydrogen peroxide assay kit. ***P < 0.001; two-tailed unpaired Student’s *t*-test. Error bars represent mean ± SD of five independent experiments

NOX5 exists and functions in the plasma membrane. Previous studies have shown that Pyk2, a scaffolding protein that exists on cell membrane,^20,21^ can be activated by hypoxia.^22^ Therefore, we evaluated whether hypoxia can induce the interaction between endogenous Pyk2 and NOX5 using immunoprecipitation assay. As shown in Fig. 3a, hypoxia facilitated the formation of NOX5/Pyk2 complex on cell membrane.

Pyk2 activation, majorly phosphorylated at Tyr^402^ (Y402) site, is dependent on the intracellular Ca^2+^. The observations in Fig. 3b and c showed that hypoxia was able to increase Ca^2+^ level (Fig. 3b) and induce the Pyk2 Tyr^402^ phosphorylation (Fig. 3c) in ESCC cells. Ca^2+^ chelator-BAPTA-AM (10 μM, pretreated with 30 minutes) inhibited the Pyk2 Tyr^402^ phosphorylation induced by hypoxia (Fig. 3c). BAPTA-AM effectively inhibited the Pyk2/NOX5 interaction on cell membrane in control vector, NOX5 or Pyk2-overexpressing KYSE30 cells, especially under hypoxic stimulation (Fig. 3d). Figure 3e showed that Pyk2 Y402F mutant blocked the Pyk2/NOX5 interaction in NOX5-overexpressing KYSE30 cells, and produced the similar effect as BAPTA-AM treatment on Pyk2/NOX5 interaction in ESCC cells.

NOX5 produces both superoxide (O_2_^.−^) and H_2_O_2_ in heterologous NOX5-overexpressing cells.^23^ H_2_O_2_ is critical for the regulation of signaling protein’s tyrosine phosphorylation and activity by oxidative modification of specific residues within the targeted protein kinase.^24,25^ Thus, we focused on NOX5-derived H_2_O_2_ in subsequent experiments. Figure 3f showed that hypoxia-stimulated H_2_O_2_ production in Pyk2/NOX5 complex in ESCC cells and NOX5 depletion inhibited hypoxia-induced H_2_O_2_ production. Then, the effect of BAPTA-AM and Pyk2 Y402F mutant plasmid on H_2_O_2_ production in Pyk2/NOX5 complex was evaluated. The results in Fig. 3g clearly showed that BAPTA-AM and Pyk2 Y402F mutant effectively downregulated the H_2_O_2_ production in Pyk2/NOX5 complex under hypoxic stimulation. These results suggest that Ca^2+^-activated Pyk2 Tyr^402^ phosphorylation is responsible for hypoxia-induced interaction of Pyk2 and NOX5 on cell membrane and subsequent stimulation of NOX5 activity in Pyk2/NOX5 complex.

### Pyk2 recruits c-Abl to stimulate NOX5 activity in Pyk2/NOX5 complex

**Fig. 4:**
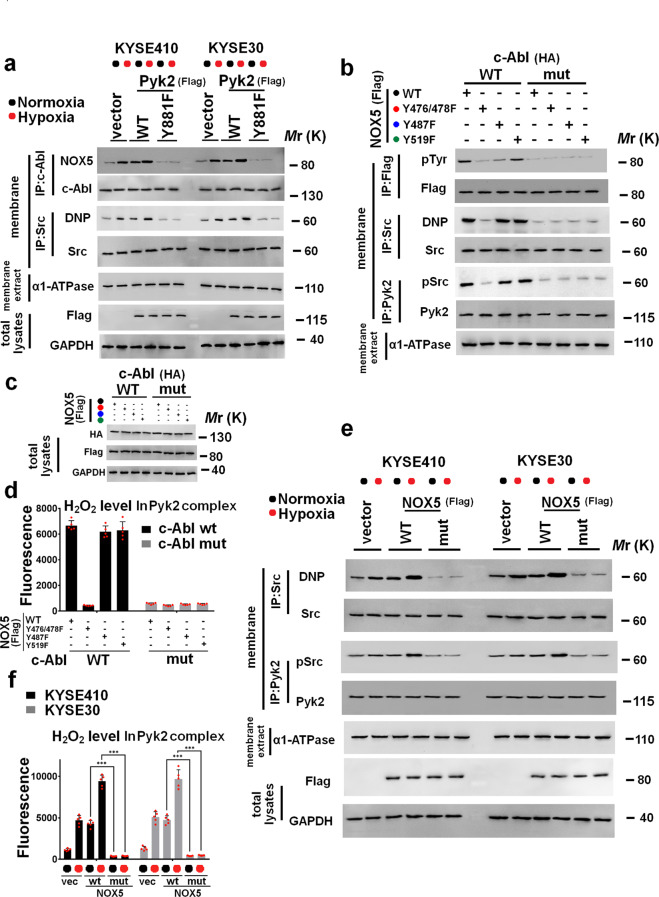
Pyk2 recruits c-Abl to enhance NOX5 activity in Pyk2/NOX5 complex. **a** KYSE30 or KYSE410 cells were transfected with vector, Flag-Pyk2 wild type (wt), or Flag- Pyk2 Y881F mutant plasmid and then cultured under normoxia or hypoxia for 1 h. Cell membrane lysates were immunoprecipitated with c-Abl antibody. Immunocomplexes were then immunoblotted using NOX5 and c-Abl antibodies (**a**). Membranous Src was immunoprecipitated with an anti-Src antibody. Oxidized Src levels were measured using a modified OxyBlot protein detection kit (**a**). The efficacy of membrane protein extraction was evaluated using immunoblotting to detect the expression of α1-ATPase (membrane biomarker) (**a**) in cell membrane lysis. Cell membrane lysates were immunoprecipitated with Pyk2 antibody. **b–d** HeLa cells were co-transfected HA-c-Abl wt or kinase-dead (KD) K290R mutant with Flag-tagged NOX5 wt, Y476/478F, Y487F, or Y519F plasmid, respectively. Cell lysates were immunoprecipitated with the antibody against Flag. Cell membrane lysates were then immunoblotted using antibodies against flag and phosphotyrosine. Membranous Src was immunoprecipitated with an anti-Src antibody. Oxidized Src levels were assayed using a modified OxyBlot protein detection kit (**b**). The transfection efficacy was measured using immunoblotting (**c**). The Pyk2 complex-derived H_2_O_2_ was tested using an Amplex red hydrogen peroxide assay kit (**d**). **e**, **f** The indicated ESCC cells were transfected with control vector, Flag-NOX5 wt, or Flag-NOX5 Y476/478F (mutant) plasmid, respectively, and then cultured under normoxic or hypoxic condition for 1 h. Oxidized Src levels were measured using a modified OxyBlot protein detection kit (**e**). The phosphorylation of Src Tyr^419^ in Pyk2 complex was immunoprecipitated with the antibody against Pyk2 and then immunoblotted using antibodies against Pyk2 and pSrc (Tyr^419^) (**e**). The efficacy of membrane protein extraction was evaluated using immunoblotting to detect the expression of α1-ATPase (membrane biomarker) (**e**) in cell membrane lysis. The Pyk2 complex-produced H_2_O_2_ was assayed using an Amplex red hydrogen peroxide assay kit (**f**). ***P < 0.001; two-tailed unpaired Student’s *t*-test. Error bars represent mean ± SD of five independent experiments

c-Abl interacts with the C-terminal domain of Pyk2 Tyr^881,,26^ and stimulates NOX5 activity.^27,28^ However, whether c-Abl stimulation of NOX5 activity is dependent on its interaction with Pyk2 remains undefined. As shown in Fig. 4a, we found that hypoxia greatly promoted interaction between NOX5 and c-Abl in ESCC cells. Introduction of Pyk2 wild type (wt) plasmid into ESCC cells enhanced NOX5/c-Abl interaction, especially under hypoxic condition (Fig. 4a). Furthermore, we observed that Hypoxia-induced NOX5/c-Abl interaction was impaired in ESCC cells expressing Pyk2 Y881F mutant (Fig. 4a). H_2_O_2_ production in Pyk2/NOX5 complex was increased or decreased in ESCC cells harboring Pyk2 wt plasmid or Y881F mutant (Supplementary Fig. 2a).

NOX5 activity is dependent on the phosphorylation of its catalytic domain.^29–31^ To determine the phosphorylating effect of c-Abl on the C-terminal catalytic domain of NOX5, HeLa cells were co-transfected with c-Abl wt, or kinase dead (KD) K290R mutant plasmid with NOX5 wt, Y476/478F, Y487F, or Y519F mutant plasmid, respectively. As shown in Fig. 4b and c, c-Abl-phosphorylated NOX5 was mostly inhibited when c-Abl plasmid co-transfected with NOX5 Y476/Y478F mutant, then the NOX5 Y487F mutant, but not with NOX5 Y519F mutant. c-Abl KD mutant was not able to induce the tyrosine phosphorylation in NOX5 (Fig. 4b, c). Similar results were also observed in the H_2_O_2_ production in Pyk2/NOX5 complex (Fig. 4d). Collectively, these results indicate that Pyk2 may act as a scaffolding protein for c-Abl stimulation of NOX5 activity in Pyk2/NOX5 complex, and c-Abl-enhanced NOX5 activity is mainly dependent on phosphorylation of NOX5 Tyr^476/478^ sites.

### Src oxidation and activation in Pyk2/NOX5 complex is dependent on NOX5 activity

It has been reported that ROS promotes Src oxidation and activation.^24^ Thus, we speculated that NOX5-derived ROS oxidized Src to stimulate its activation in ESCC cells. When NOX5 was depleted, hypoxia did not apply evident effect on Src oxidation and activation in Pyk2/NOX5 complex (Supplementary Fig. 3). After Pyk2 wt plasmid was transfected into ESCC cells, Src oxidation and activation in Pyk2/NOX5 complex were substantially enhanced, whereas Pyk2 Y881F mutant abrogated these effects (Fig. 4a and Supplementary Fig. 2b). Furthermore, c-Abl plasmid co-transfected with NOX5 wt plasmid could effectively oxidize Src in HeLa cells, but not with NOX5 Y476/Y478F mutant (Fig. 4b, c). Src oxidation in Pyk2/NOX5 complex was significantly impaired in HeLa cells harboring c-Abl KD mutant in the presence of NOX5 wt and Y476/478F, Y487F, or Y519F mutant (Fig. 4b). The similar results were also obtained in Src activation in Pyk2/NOX5 complex in HeLa cells (Fig. 4b). NOX5 Y476/478F mutant suppressed the H_2_O_2_ production in Pyk2/NOX5 complex, Src oxidation and activation (Fig. 4e, f, and Supplementary Fig. 4a, b) in ESCC cells, especially under hypoxic condition. Taken together, these results indicate that NOX5 oxidizes and activates Src in Pyk2/NOX5 complex via c-Abl dependent manner.

### NOX5/Src axis promotes ESCC progression

**Fig. 5:**
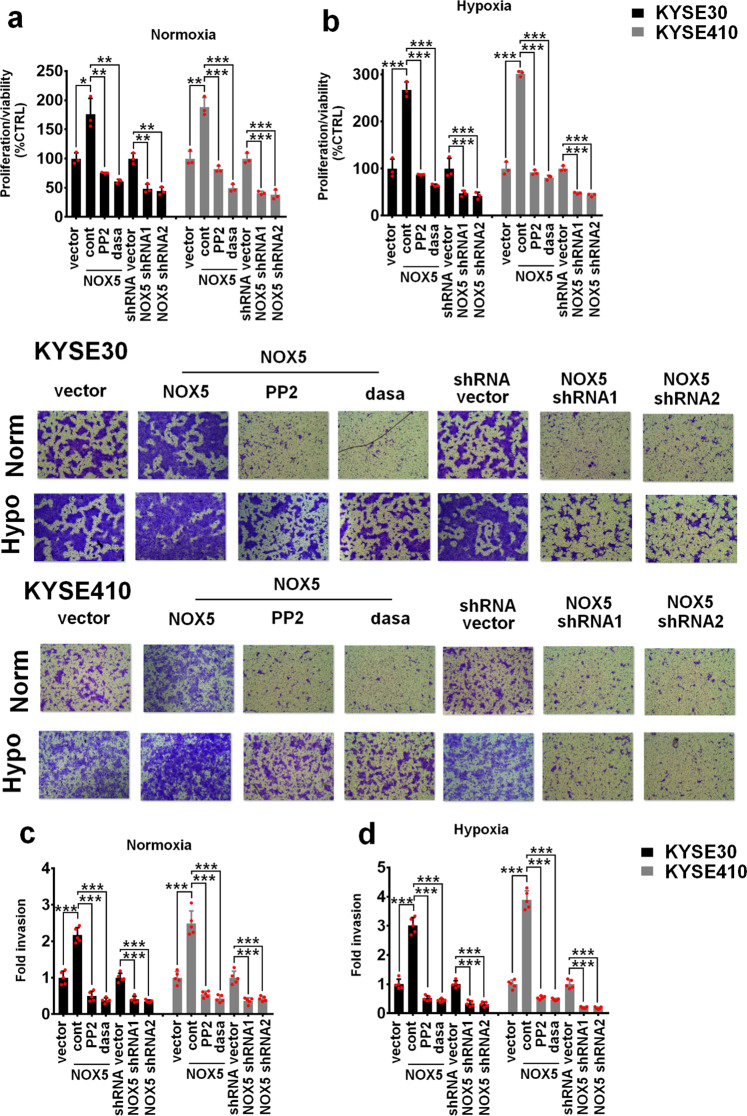
NOX5 promotes ESCC progression in vitro. **a**, **b** NOX5-overexpressing KYSE30 (**a**) or KYSE410 (**b**) cells were treated with 100 nM dasatinib, 1 μM PP2 or control solvent, or scramble control or NOX5-silenced KYSE30 or KYSE410 cells were cultured under normoxic and hypoxic conditions for 3 days. The cell growth was measured by MTS assay. **c**, **d** above cells were cultured under normoxic and hypoxic conditions for 24 h. The tumor invasion was evaluated by Transwell invasion assay. *P < 0.05; **P < 0.01; ***P < 0.001; two-tailed unpaired Student’s *t*-test. Error bars represent mean ± SD of three or five independent experiments

We further evaluated whether NOX5 could confer tumor cell ability of aggressiveness. The MTS assay showed that NOX5 stably overexpressing in ESCC cells resulted in significantly higher growth rates compared with control cells under either normoxic or hypoxic conditions (Fig. 5a, b). Overexpression of NOX5 also strongly promoted the invasive ability of ESCC cells (Fig. 5c, d). In contrast, stable depletion of NOX5 substantially suppressed cellular growth and decreased the invasive ability of ESCC cells, especially under hypoxia (Fig. 5a–d). Stable transfection of NOX5 Y476/Y478F mutant into ESCC cells inhibited ESCC malignant progression in vitro (Supplementary Fig. 4c–f). We also determined whether Src was involved in NOX5-promoted ESCC progression. As shown in Fig. 5a–d, the addition of Src inhibitor (dasatinib or PP2) was able to attenuate NOX5-promoted ESCC malignant progression in vitro.

**Fig. 6:**
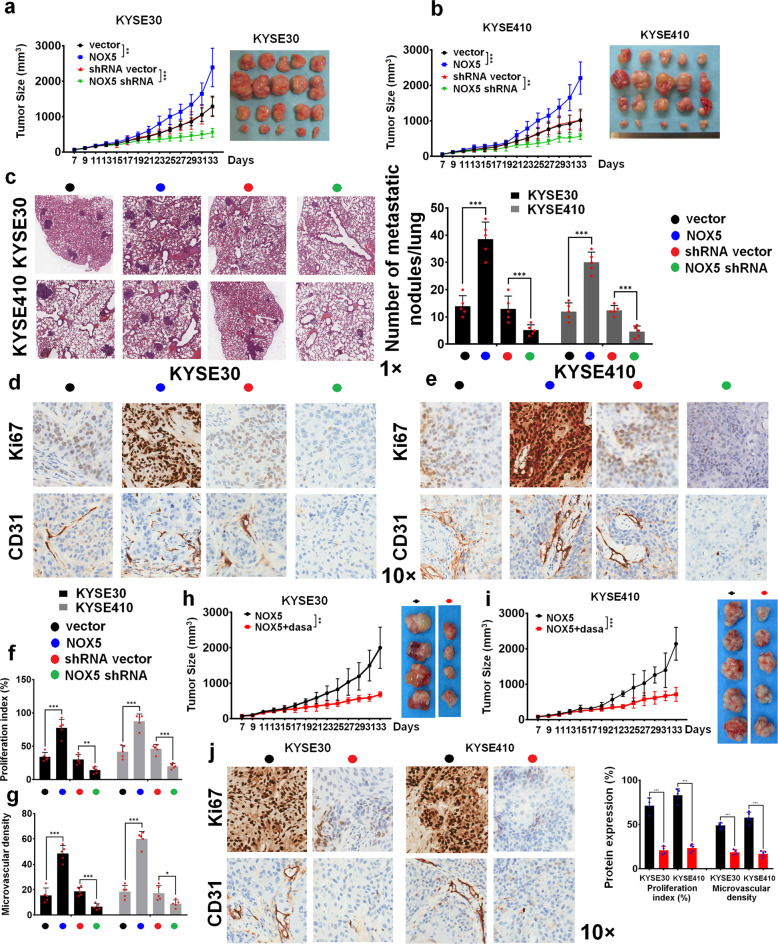
NOX5 promotes ESCC progression in vivo. **a**, **b** KYSE30 (**a**) and KYSE410 (**b**) cells stably expressing NOX5 or shNOX5 or their respective control vector were subcutaneously inoculated into mice (*n* = 5 biologically independent mice per group). The growth curves and representative images of tumor were shown. **c** A lung colonization model was established in mice by injecting intravenously with the indicated cells via lateral tail veins (*n* = 5 biologically independent mice per group). Representative H&E staining of lungs and the number of metastatic nodes on the surface of the lungs were shown. Magnification, ×1 as indicated. **d**, **e** IHC analysis of Ki-67 and CD31 in KYSE30 (**d**) and KYSE410 (**e**) tumors stably expressing NOX5 or shNOX5 or their respectively control vector. Magnification, ×10 as indicated. **f**, **g** Statistical analyses of the expression of Ki-67 (**f**), or CD31 (**g**) in the tumor tissues of KYSE30 (left panel) and KYSE410 (right panel) cells stably expressing NOX5 or shNOX5 or their respective control vector. **h**, **i** KYSE30 (**h**) (*n* = 4 biologically independent mice per group) and KYSE410 (**i**) (*n* = 5 biologically independent mice per group) cells stably overexpressing NOX5 plasmid were subcutaneously inoculated into mice treated with dasatinib (30 mg/kg/day, per os) or control solvent. The growth curves and representative images of tumor were shown. **j** IHC analyses of the expression of Ki-67, or CD31 in the indicated tumor tissues. **P < 0.01; ***P < 0.001; two-tailed unpaired Student’s *t*-test. Error bars represent mean ± SD of five independent experiments

To extend our in vitro observations, we investigated whether NOX5 could regulate tumor growth in vivo using the subcutaneous xenograft model. As shown in Fig. 6a and b, the tumors derived from ESCC/NOX5 cells grew faster than vector-control tumors. Conversely, the xenografts formed by NOX5-depleted cells revealed slower growth than control tumors. Furthermore, we examined the effect of NOX5 on ESCC progression in a lung colonization model. Nude mice were intravenously injected with NOX5-transduced or NOX5-depleted cells and their respective control cells via lateral tail veins. As shown in Fig. 6c, the numbers of tumor nests in the lungs formed by NOX5-transduced cells was significantly higher compared with control cells, whereas depletion of NOX5 resulted in the lower numbers of tumor nests in the lungs compared with control cells, indicating that NOX5 effectively regulates the lung colonization of ESCC cells. IHC analysis demonstrated that NOX5-overexpressing tumors displayed higher Ki-67 proliferation index and CD31 microvascular density (Fig. 6d–g). On the contrary, depletion of NOX5 substantially reduced proliferation index and microvascular density (Fig. 6d–g).

Additionally, we evaluated whether inhibition of Src activity can impair NOX5-mediated tumor progression. Figure 6h, i showed that dasatinib treatment significantly retarded the growth of NOX5-overexpressing KYSE30 (Fig. 6h) or KYSE410 (Fig. 6i) tumor in vivo. As shown in Fig. 6j, in NOX5-overexpressing ESCC tumors, inhibition of Src activity effectively reduced the expression of Ki-67 and CD31. The tumors derived from NOX5 Y476/Y478F mutant-stably transfected ESCC cells grew slower and showed lower expression of Ki-67 and CD31 than control tumors (Supplementary Fig. 5a–g). Consistent with the results from the in vitro study, these observations indicate that NOX5-induced ESCC progression is mainly dependent on the activation of Src.

## Discussion

Whereas hypoxia-stimulated ROS promoting tumor malignant progression has been extensively studied, it is still unclear how hypoxia-stimulated ROS axis involves in tumor progression.^1–3^ In this study, we attempt to establish the clinical relationship between ROS producer-NOXs and hypoxia in ESCC. Our results demonstrate that NOX5 is tightly related with hypoxia and its expression is positively correlated with malignant phenotypes and shorter survival in ESCC patients clinically. Using genetic modulation of NOX5 in ESCC cells, we demonstrate that NOX5 effectively promotes progression of ESCC cells both in vitro and in vivo. Importantly, we find that the assembly of NOX5-based signalosome can enhance the NOX5 activity on cell membrane and subsequently concentrate the local ROS to oxidize and activate oncoprotein-Src.

Although ROS produced by NOXs participate in signal transduction, the mechanisms by which stresses or protein receptors activate NOXs and regulate ROS generation are poorly understood. Pyk2 is activated by an increase intracellular Ca^2+^, which could be caused by a variety of extracellular stimuli, especially hypoxia. After stimulation, Pyk2 undergoes auto-phosphorylation at Tyr^402^ site, leading to recruit and activate Src family kinases (SFK) or other protein kinases, indicating that Pyk2 functions as the hub of extracellular stress and intracellular signaling pathways.^32–34^ Our study finds that hypoxia stimulates intracellular Ca^2+^ to induce Pyk2 Tyr^402^ phosphorylation, accompanied by membranous interaction of Pyk2/NOX5, which is critically contributed to the induction of NOX5 activity. These results suggest that Ca^2+^-mediated Pyk2 phosphorylation may possibly change Pyk2 conformation to facilitate the membranous interaction of Pyk2 and NOX5. Previous studies have shown that Ca^2+^ directly binds to NOX5 and enhances its activity.^35^ Besides, Ca^2+^ activates calmodulin-dependent kinase II (CAMK II) to phosphorylate NOX5 Ser^475^ site to stimulate its activity in COS-7 cells.^30^ The present study uncovers a new mechanism of Ca^2+^-induced NOX5 activity involves enhancing phosphorylation of Ca^2+^-sensitive protein-Pyk2 to induce the membranous interaction of Pyk2/NOX5. However, the mechanism of hypoxia-induced intracellular Ca^2+^ elevation is complicated^36^ and it is unclear how hypoxia stimulates Ca^2+^ channel or other proteins to upregulate intracellular Ca^2+^ in tumor cells. Thus, the origin of the elevated intracellular Ca^2+^ in hypoxic tumor cells need to be further investigated in the future.

Pyk2 interacts with c-Abl,^21^ which induces NOX5 activity in K562 cells.^27^ However, the exact mechanism of Pyk2-interacted c-Abl activating NOX5 remains unclear. Phosphorylation is a key regulatory step in the activation of NADPH oxidase, especially NOX2 and NOX5.^30,37^ Previous study found that the phosphorylation of Serine or Threonine residue in NOX5 catalytic domain is required for NOX5 activity.^30^ This study has demonstrated that c-Abl interacts with NOX5 to phosphorylate the Tyr^476/478^ sites within NOX5 and subsequently enhances H_2_O_2_ production inside the Pyk2/NOX5 complex. Thus, our study has identified novel tyrosine phosphorylation sites required for NOX5 activity under certain stress. Importantly, the c-Abl/NOX5 interaction is under the control of Pyk2, of which Tyr^881^ site phosphorylation is critical for such interaction, further demonstrating that c-Abl-phosphorylated NOX5 activation is mainly dependent on the scaffold provided by Pyk2. H_2_O_2_ is critical for the regulation of signaling proteins via oxidative modification of cysteines within the targeted protein.^24,38^ However, it does not cause accidental oxidation of non-specific targets along its journey toward the intended protein. Some NOXs regulate specific redox-signaling pathways in cells through secreting ROS to the plasma membrane, endosomes, focal adhesion, or other cellular compartments, and then activate or suppress local oncoproteins or tumor suppressors.^8,39^ Our data show that the formation of Pyk2/NOX5/c-Abl complex facilitates redox-sensitive activation of Src at focal complex. This biological event, in turn, may possibly promote tumor malignant progression.

**Fig. 7:**
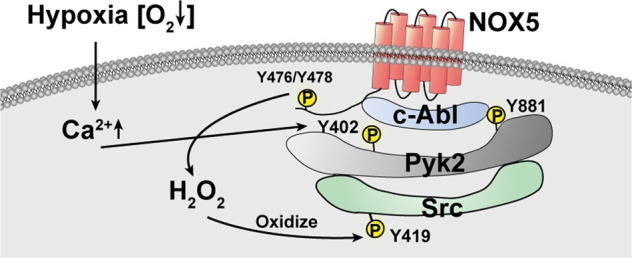
Proposed Model of Membranous NOX5-derived local ROS oxidizes and activates Src to promote growth and invasion of ESCC cells. Hypoxia stimulated the interaction between NOX5 and Pyk2 on cell membrane via enhancing intracellular Ca^2+^-mediated Pyk2 Tyr^402^ phosphorylation. Subsequently, Pyk2 acted as a scaffold via its Tyr^881^ site for c-Abl phosphorylating the catalytic domain of NOX5 Tyr^476/478^ and then upregulated H_2_O_2_ inside the Pyk2/NOX5 complex to oxidize and activate Src

It remains to be further investigated whether other protein kinases can be oxidatively modified by ROS during tumor progression. Also relevant to our observations, it will also be important to determine how NOXs-derived ROS accurately oxidize the local protein kinases in specific cellular compartments. Importantly, our study characterizes the possible mechanisms of NOXs-upregulated ROS in tumor cells under hypoxic condition and potentially allows the development of a novel diagnostic marker and therapeutic strategy for gastrointestinal cancers (Fig. 7).

## Materials and methods

### Reagents and antibodies

Antibody against NOX5 was from Abnova (Cat# PAB17793), antibodies against Ki-67 (Cat# 9027), CD31 (Cat# 3528), GAPDH(Cat# 5174), Flag (Cat# 2368), HA (Cat# 2367), c-Abl (Cat# 2862), Src (Cat# 2108) were from CST, antibodies against Pyk2 (Cat# ab226798), α1-ATPase (Cat# ab7671), phosphotyrosine (Cat# ab10321), DUOX2 (Cat# ab97266), Carbonic anhydrase IX (CA IX) (Cat# ab15086) were from Abcam, pSrc (Tyr^419^) (Cat# 102-17936) was from Raybiotech, NOX1 (Cat# A8527), NOX2 (Cat# A1636), NOX3 (Cat# A3677), NOX4 (Cat# A11904), DUOX1 (Cat# A8583) were obtained from ABclonal, Src inhibitors-dasatinib (Cat# S1021) and PP2 (Cat# S7008) were purchased from Selleck Chemicals. PEG-catalase (Cat# C4963-2MG), Diphenyleneiodonium (DPI; Cat# D2926-10MG), and N-Acetyl-L-cysteine (NAC; Cat# A9165-5G) were purchased from Sigma-Aldrich.

### Cell lines and hypoxic treatment

The human ESCC cell lines, KYSE140, KYSE150, KYSE180, KYSE30, KYSE410, KYSE450, KYSE510, KYSE70, and Eca109 (generously provided by Dr Shemada of Kyoto University), were originally maintained in RPMI 1640 medium supplemented with 10% fetal bovine serum (FBS), penicillin (100 U/ml), and streptomycin (100 μg/ml). HeLa cell line was purchased from the Cell Bank of Type Culture Collection of Chinese Academy of Sciences, Shanghai institute of Biochemistry and Cell Biology. All cell lines were recently authenticated by cellular morphology and the short tandem repeat analysis using the AmpF/STR Identifier Kit (Applied Biosystems). Primarily cultured NEECs were obtained from CHI Scientific, Inc. Primary esophageal squamous cell carcinoma (ESCC) cells were isolated from two cases of freshly removed tumor samples using Cell Isolation Kit (Panomics) according to the manufacturer’s instruction. All cells were free of mycoplasma infection. Cells were maintained at 37 °C in a humidified 95% air and 5% CO_2_ incubator. For hypoxic treatment, cells were placed in a modulator incubator (Thermo Electron Co.) in an atmosphere consisting of 94% N_2_, 5% CO_2_ and 1% O_2_.

### Plasmids and transfection

For establishing stable NOX5 shRNA cell lines, KYSE30 and KYSE410 cells were transfected with the pLKO.1 (Addgene) and pLKO.1 vector expressing shRNA for NOX5 knockdown (pLKO.1-shNOX5). Transfected cells were selected by 0.5 μg/ml puromycin for 10–14 days. Positive clones were selected and amplified for further analyses. The NOX5 shRNA sequences used in the present study as follows: shRNA1-CAAGTCCTACCACTGGACCAGAACATCCA and shRNA2-TGGCTCACACTGTGAACTTTGTACTCCAG. Stable cell lines expressing NOX5 were generated by transfection of pMSCV (Clontech)/NOX5 into KYSE30 and KYSE410 cells and then cultured for 10–14 days with 0.5 μg/ml puromycin. Stable cell lines expressing Flag-NOX5 Y476/478F mutant were generated by transfection of pcDNA3.1/ Flag-NOX5 Y476/478F into KYSE30 and KYSE410 cells and then cultured for 10–14 days with 400 μg/ml G418. Positive clones were selected and amplified for further analyses. Various deletion mutants of Flag-tagged NOX5 or Pyk2 and HA-tagged NOX5 or Pyk2 were cloned into a pcDNA3.1 vector. Mutations of NOX5 (Flag-tagged NOX5 Y476/478F, Y487F, Y519F), Pyk2 (Flag-tagged Pyk2 Y402F, Y881F), or c-Abl (HA-tagged c-Abl K290R) were introduced into the indicated sites using the Phusion^TM^ high-fidelity DNA polymerase (Thermo Fisher; Cat# F530L) and then cloned into a pcDNA3.1 vector.

### Cell proliferation/viability assay

The indicated cells were seeded in a 96-well plate under normoxia or hypoxia, and then treated with the indicated inhibitors for 3 days. The cell growth rate was determined using the MTS assay (Promega) according to the manufacturer’s instructions. Experiments were performed three times, with duplicate replicates.

### Invasion assay

The transwell invasion assay was performed using the transwell chamber with a Matrigel-coated filter. The indicated cells to be tested were starved in serum-free medium for 12 h and then plated on the top chamber with or without the indicated agents under normoxic or hypoxic condition for 24 h, followed by removal of cells inside the upper chamber with cotton swabs, and the invasive cells on the lower side were fixed, stained with 0.1% crystal violet solution, and counted using light microscope. The experiment was repeated five times.

### Immunoblotting

Immunoblotting was performed according to the protocol of a standard described previously.^40^ In brief, protein extracts were loaded and electrophoresed on 8–12% SDS gel and transferred to polyvinylindene fluoride (PVDF) or nitrocellulose filter (NC) membranes. The membranes were subsequently probed with primary antibodies, respectively. All of the first antibodies were diluted at 1:1000 except for GAPDH (loading control) at 1:5000. Antibody binding was detected by enhanced chemiluminescence detection kit (ECL) (UK Amersham International plc).

### Cell membrane extraction and immunoprecipitation

For immunoprecipitation assays, cells were washed with cold PBS and lysed with 400 μl hypotonic buffer (Amresco). The lysates were incubated on ice for 30 min and then centrifuged at 10000 g at 4 °C for 20 min. The supernatant was cytosolic extract. Pellets were resuspended in 200 μl extraction buffer (20 mM HEPES (pH 7.4), 150 mM NaCl, 1 mM EDTA, 1% Triton X-100, 10% glycerol, 1 μg/ml leupeptin, 1 μg/ml aprotinin, 1 mM PMSF, and 1 mM sodium orthovanadate) and then centrifuged at 15000 g at 4 °C for 10 min to obtain supernatants containing membrane extracts. The extraction efficiency of membranous components was evaluated using immunoblotting to detect the expression of α1-ATPase (membranous marker; 1:1000). Cellular membranous extracts were incubated with appropriate primary antibodies, including anti-Pyk2 (1:100), anti-c-Abl (1:100), and protein A/G sepharose beads (ThermoFisher Scientific) on a rotator at 4 °C overnight. The immune complexes were subjected to SDS-PAGE followed by immunoblotting. Immunodetection was performed using enhanced chemiluminescence according to the manufacturer’s instructions.

### Intracellular Ca^2+^ evaluation

For evaluation of intracellular Ca^2+^ level, cells were cultured in six-well plates at 80% confluence and grown under the indicated condition. Then, cells were harvested and suspended in 500 μl calcium assay buffer and put on ice for quickly pipetting a few times, and subsequently centrifuged at 15000 g at 4 °C for 10 min to obtain supernatants. Ca^2+^ level in the supernatants was evaluated using the Calcium detection assay kit (Abcam; catalog# ab102505) according to the manufacturer’s instruction. The experiment was repeated five times.

### ELISA assay

For measurement of activated Src (pSrc Tyr^419^/Src ratio) and Pyk2 (pPyk2 Tyr^402^/Pyk2 ratio), cells were cultured in 12-well plates at 80% confluence and grown under the indicated condition. Levels of activated Src and Pyk2 in the cell lysis (~20 μg protein per sample) were measured using the human phosphor-Src (Tyr^419^) ELISA kit (Raybiotech; catalog# PEL-SRC-Y419-T) and the human phosphor-Pyk2 (Tyr^402^) ELISA kit (Raybiotech; catalog# PEL-PYK2-Y402-T) following the manufacturer’s protocols. The activation status of Src and Pyk2 was evaluated according to the formula that the optical density (OD) value of pSrc and pPyk2 /the OD value of total Src and Pyk2. The experiment was repeated three or five times.

### Src protein oxidation assay

Src oxidation was measured using a modified OxyBlot protein detection kit (Abcam; catalog# ab178020) according to the manufacturer’s instruction. After the immunoprecipitation of membranous Src (1:100), the immune complex was released from the protein A/G sepharose beads and derivatized with 20 μl 1 × 2, 4-dinitrophenylhydrazine (DNPH) solution at room temperature for 15 min. The reaction was stopped by adding 15 μl neutralization solution and separated by a SDS-PAGE gel and then subjected to immunoblotting. The oxidized Src was detected by using 1x primary anti-DNP antibody in blocking buffer overnight at 4 °C.

### Measurement of H_2_O_2_ level

The concentration of H_2_O_2_ in Pyk2/NOX5 complex was evaluated using previously reported method with a few modifications.^11^ Briefly, after the immunoprecipitation of membranous Pyk2 (1:100), the immune complex was incubated with 500 μl of 50 mM phosphate buffer (pH7.4, 1 mM EGTA, 150 mM sucrose, and 100 μM NADPH) for 1 hour at room temperature. The above procedures were completed in anaerobic condition. Then, H_2_O_2_ was measured using an Amplex red hydrogen peroxide assay kit (Thermo Scientific; catalog# A22188) following the manufacturer’s instruction. The experiment was repeated five times.

### Antibody array

For the phospho-kinase activation study, antibody arrays against 43 kinase phosphorylation sites (R&D systems; catalog# ARY003) were used according the manufacturer’s instruction. Cell lysates (1 ml) were added to each membrane. Spot quantization was performed using enhanced chemiluminescence according to the manufacturer’s instructions, with a fixed volume size for all spots being compared, and mean densities were calculated for each spot in duplicate.

### Patient information, tissue specimens, and immunohistochemistry

Tissue specimens were collected from ESCC patients with approval from the Institutional Review Board of Peking University Cancer Hospital & Institute. Inclusion criteria were patients with ESCC, having tumor stages I to III, or having having complete clinicopathologic data, which included age, sex, histopathologic diagnosis and pathologic tumor stages, and receiving surgery as initial treatment modality. For IHC assay, the tissue sections were deparaffinized, soaked in 10 μM Tris-EDTA buffer (pH 9.0) and boiled in the autoclave for 15 min to retrieve cell antigens. The primary antibodies were applied to the slides and incubated at 4 °C overnight. The primary antibodies used for clinical IHC assay were: anti-NOX1 (1:100), anti-NOX2 (1:100), anti-NOX3 (1:100), anti-NOX4 (1:100), anti-NOX5 (1:100), anti-DUOX1 (1:100), anti-DUOX2 (1:100), anti-CA IX (1:1000), or anti-pSrc (1:200). The staining index (SI) was calculated as staining intensity score × proportion of positive tumor cells.^41^ Cutoff values for high- and low-expression of proteins of interest were chosen based on a measurement of heterogeneity using the log-rank test with respect to overall survival and the chi-square test to analyze correlations between two factors.

### Animal experiments

The tumor growth of KYSE30 and KYSE410 scramble cont shRNA, KYSE30 and KYSE410 NOX5 shRNA cells, KYSE30 and KYSE410 cont vector, KYSE30 and KYSE410 NOX5-overexpressing cells, or KYSE30 and KYSE410 NOX5 Y476/478 F mutant cells (3.5 × 10^6^ cells per cell line; (*n* = 5 biologically independent mice per group) was determined following subcutaneous injection of cells into the right flank of 5-week-old female nude mice (nu/nu mouse, Vital River Laboratories, Beijing, China). For evaluating the effect of NOX5/Src axis on tumor growth, KYSE30 (*n* = 4 biologically independent mice per group) and KYSE410 (*n* = 5 biologically independent mice per group) NOX5 overexpressing cells were subcutaneously injected into the right flank of mice. Dastatinib treatment (30 mg/kg/day, *per os*) was initiated when tumors reached 80–100 mm^3^. Tumor sizes were calculated according to the formula: (mm^3^) = (L × W^2^) × 0.5. In all, 5 μm sections were cut and subjected to IHC staining. After deparaffinization, sections were IHC analysis using an anti-Ki67 (1:1000), or anti-CD31 (1:100) antibody.

The metastatic ability of above cancer cells (1 × 10^6^ cells per cell line) in vivo was observed following intravenous injection of cells into the tail vein of nude mice (*n* = 5 biologically independent mice per group). After 8 weeks, the mice were sacrificed and the number of metastatic nodules on the lung surface was counted. Metastatic lungs were fixed with 4% paraformaldehyde before dehydration and paraffin embedding. Paraffin sections were stained with hematoxylin and eosin (H&E) according to standard protocols. Animal handling and procedures were ethically approved by the Animal Center, Peking University Cancer Hospital & Institute.

### Statistical analysis

All statistical analyses were performed using Graphpad 7.0 software and MATLAB7.0 software. Student’s *t*-test (two-tailed) was used to compare statistical significance between two groups. The chi-square test was employed to analyze correlations between two factors. The overall survival curves were established by the Kaplan–Meier method, and significant differences were compared using the log-rank test. All bars represent the mean ± SD derived from three to five independent experiments. *P*-value of <0.05 was considered statistically significant. All experiments were performed three to five independently under similar conditions, unless otherwise specified in the figure captions.

## Supplementary information

supplementary Figure and Table legends

supplementary Figure 1

supplementary Figure 2

supplementary Figure 3

supplementary Figure 4

supplementary Figure 5

supplementary Table 1

supplementary Table 2

## Data Availability

All data supporting the findings of this study are available from the corresponding author on reasonable request.
