# Supplementary material for: Membranous NOX5-derived ROS oxidizes and activates local Src to promote malignancy of tumor cells
Source: Signal Transduct Target Ther. 2020 Aug 14;5:139. doi: 10.1038/s41392-020-0193-z (PMC7426961; doi:10.1038/s41392-020-0193-z)
Supplement: Supplementary file 1 — supplementary Figure and Table legends [file 41392_2020_193_MOESM1_ESM.doc]

**SUPPLEMENTARY FIGURE LEGENDS**

**Supplementary Fig. 1. .** Negative control staining of ESCC slide. Magnification, 20 × as indicated.

**Supplementary Fig. 2. a, b** The indicated cells were transfected with control, Flag-Pyk2 wt or Pyk2 Y881F plasmids, and then exposed to normoxic or hypoxic condition. The Pyk2 complex-produced H_2_O_2_ was evaluated using an Amplex red hydrogen peroxide assay kit **(a)**. The membranous interaction between Pyk2 and pSrc was examined using immunoprecipitation and immunoblotting **(b)**.

**Supplementary Fig. 3.** The KYSE30 and KYSE410 control shRNA or NOX5 shRNA cells were cultured under normoxic or hypoxic condition for 1 hour. Membranous Src was immunoprecipitated with an anti-Src antibody. Oxidized Src levels were measured using a modified OxyBlot protein detection kit. Cell membrane lysates were immunoprecipitated with the antibody against Pyk2. Immunocomplexes were then immunoblotted using antibodies against Pyk2 and pSrc (Tyr^419^). The efficacy of membrane protein extraction was examined using immunoblotting to detect the expression of α1-ATPase (membrane biomarker) in cell membrane lysis.

**Supplementary Fig. 4. a** The KYSE30 and KYSE410 cells were stably transfected with control vector or Flag-NOX5 Y476/478F mutant plasmid. The transfection efficacy was assayed by immunnblotting. **b** The KYSE30 and KYSE410 control vector or Flag-NOX5 Y476/478F mutant cells were cultured under normoxic or hypoxic condition for 24 hours. Src activity was evaluated using quantitative ELISA assay. **c, d** The KYSE30 **(c)** and KYSE410 **(d)** control vector or Flag-NOX5 Y476/478F mutant cells were cultured under normoxic and hypoxic conditions for 3 days. The cell growth was evaluated by MTS assay. **e, f** above cells were cultured under normoxic and hypoxic conditions for 24 hours. The tumor invasion was evaluated by Transwell invasion assay. ****P*<0.001; two-tailed unpaired Student's *t*-test. Error bars represent mean ± SD of three or five independent experiments.

**Supplementary Fig. 5. a, b** KYSE30 **(a)** and KYSE410 **(b)** cells stably expressing NOX5 Y476/478F mutant or its control vector were subcutaneously inoculated into mice (n=5 biologically independent mice per group). The growth curves and representative images of tumor were shown. **c** A lung colonization model was established in mice by injecting intravenously with the indicated cells via lateral tail veins (n=5 biologically independent mice per group). Representative H&E staining of lungs and the number of metastatic nodes on the surface of the lungs were shown. Magnification, 1× as indicated. **d, e** IHC analysis of Ki-67 and CD31 in KYSE30 **(d)** and KYSE410 **(e)** tumors stably expressing NOX5 Y476/478F mutant or its respectively control vector. Magnification, 10× as indicated. **f, g** Statistical analyses of the expression of Ki-67 **(f)** and CD31 **(g)** in the indicated tumor tissues. ****P*<0.001; two-tailed unpaired Student's *t*-test. Error bars represent mean ± SD of five independent experiments.

**SUPPLEMENTARY TABLE LEDGENDS**

**Supplementary Table 1**

Expression of NOXs in human esophageal squamous cell carcinoma (esophageal squamous cell carcinoma cohort Ⅰ, n=92)

**Supplementary Table 2**

Correlation between the clinopathologic features and expression of NOX5 in esophageal squamous cell carcinoma (esophageal squamous cell carcinoma cohort Ⅱ, n=95)
