# Supplementary figures and images for: Membranous NOX5-derived ROS oxidizes and activates local Src to promote malignancy of tumor cells
Source: Signal Transduct Target Ther. 2020 Aug 14;5:139. doi: 10.1038/s41392-020-0193-z (PMC7426961; doi:10.1038/s41392-020-0193-z)

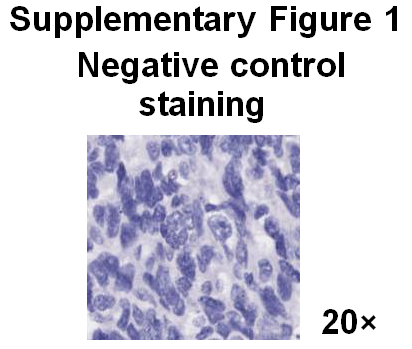

Supplement: Supplementary file 2 — supplementary Figure 1 [file 41392_2020_193_MOESM2_ESM.tif]

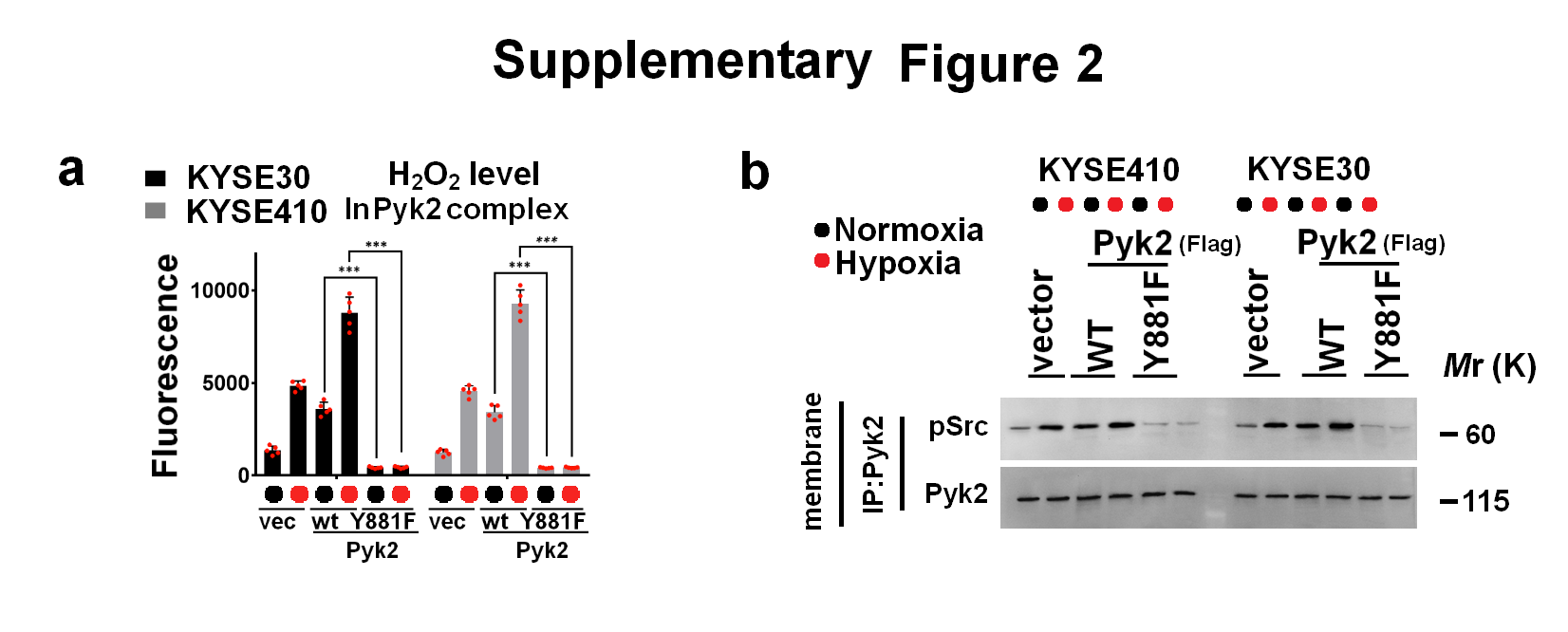

Supplement: Supplementary file 3 — supplementary Figure 2 [file 41392_2020_193_MOESM3_ESM.tif]

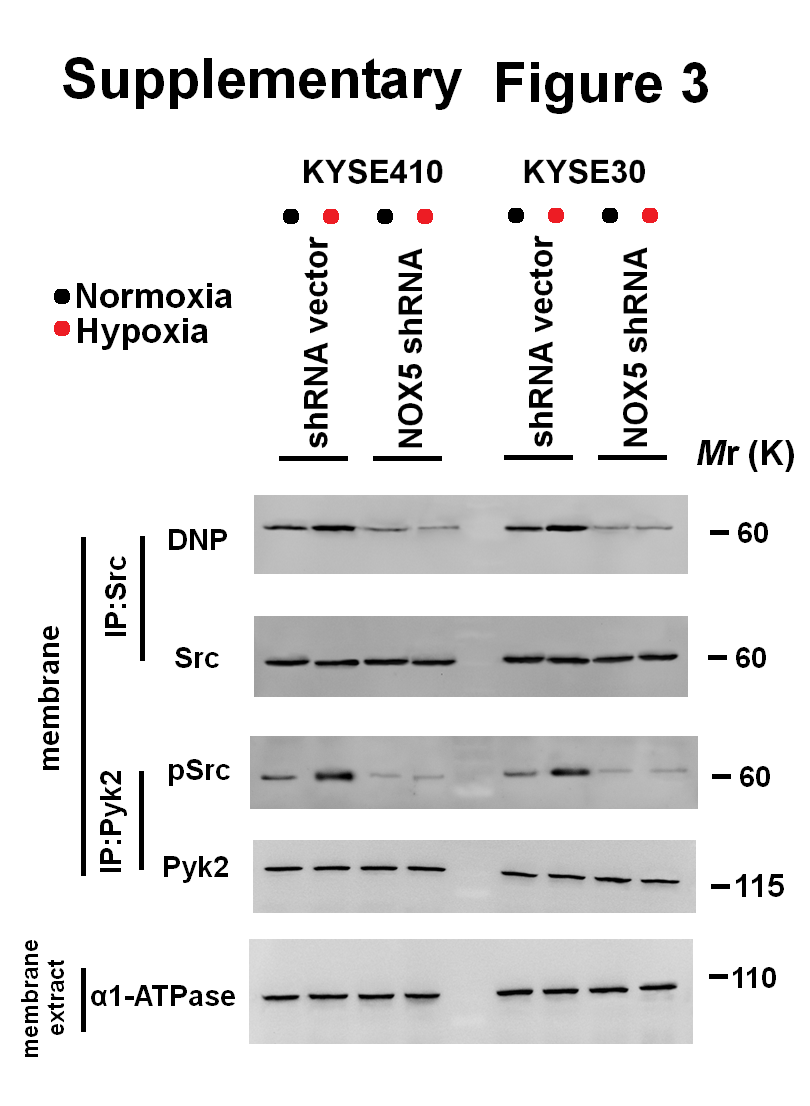

Supplement: Supplementary file 4 — supplementary Figure 3 [file 41392_2020_193_MOESM4_ESM.tif]

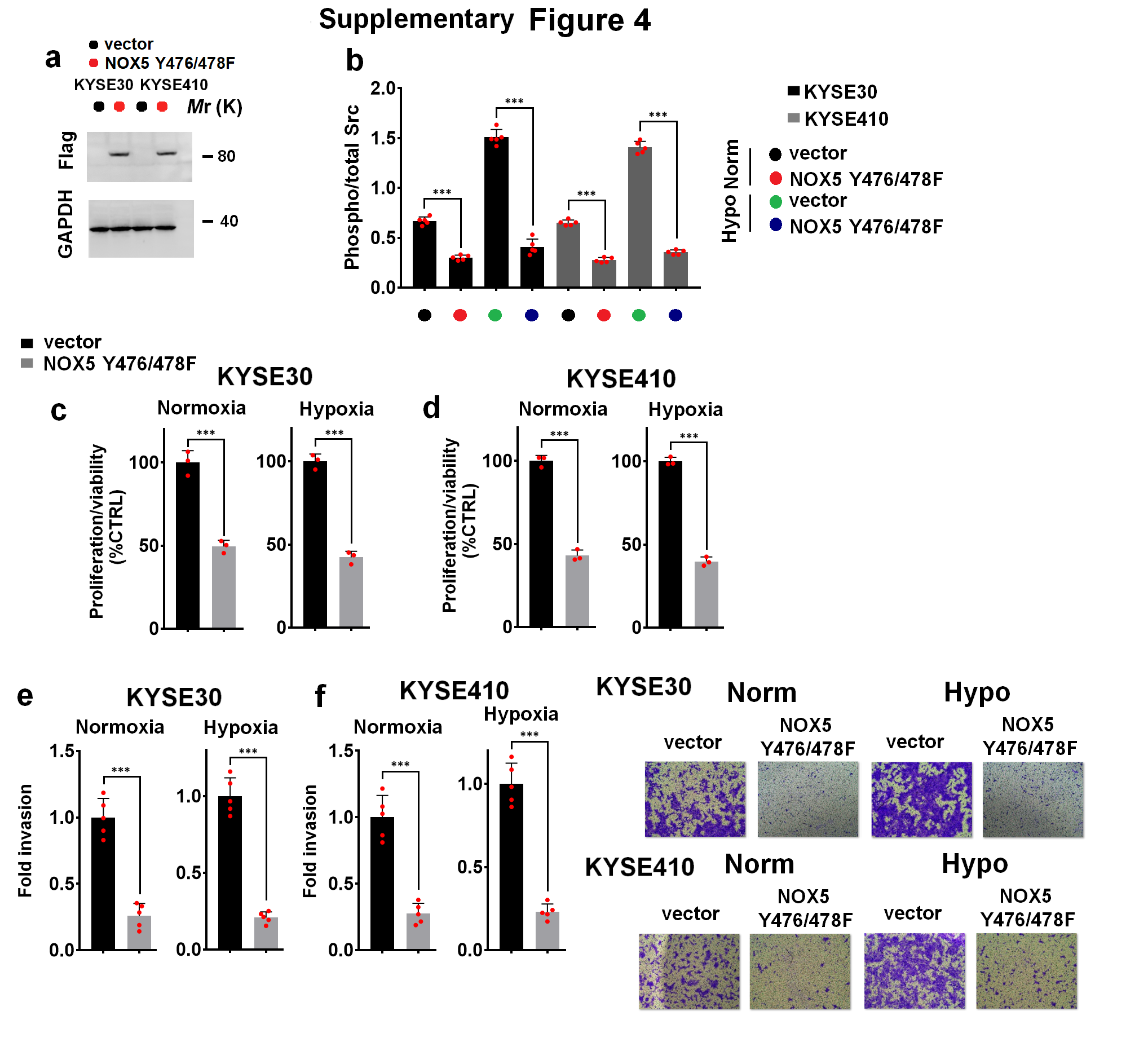

Supplement: Supplementary file 5 — supplementary Figure 4 [file 41392_2020_193_MOESM5_ESM.tif]

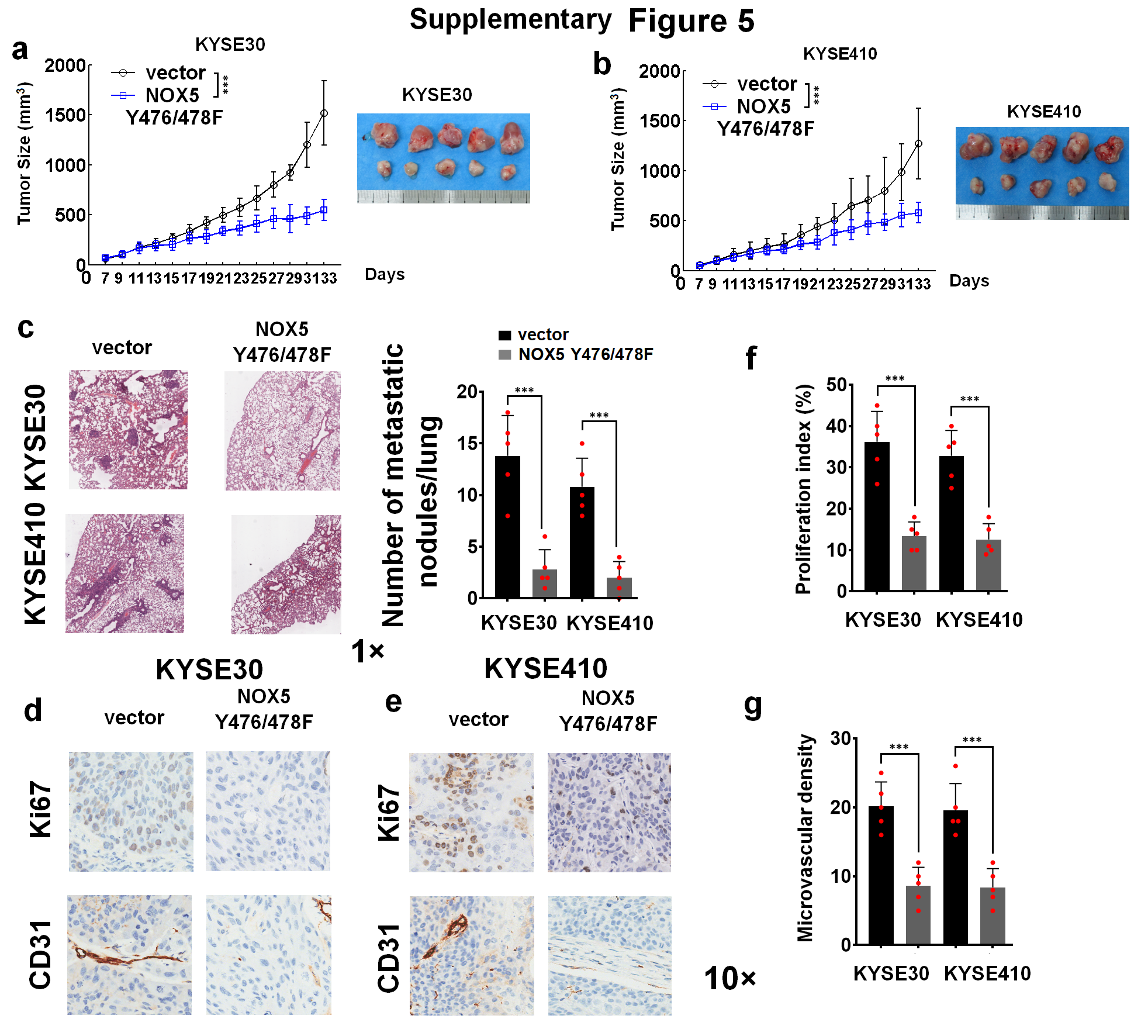

Supplement: Supplementary file 6 — supplementary Figure 5 [file 41392_2020_193_MOESM6_ESM.tif]

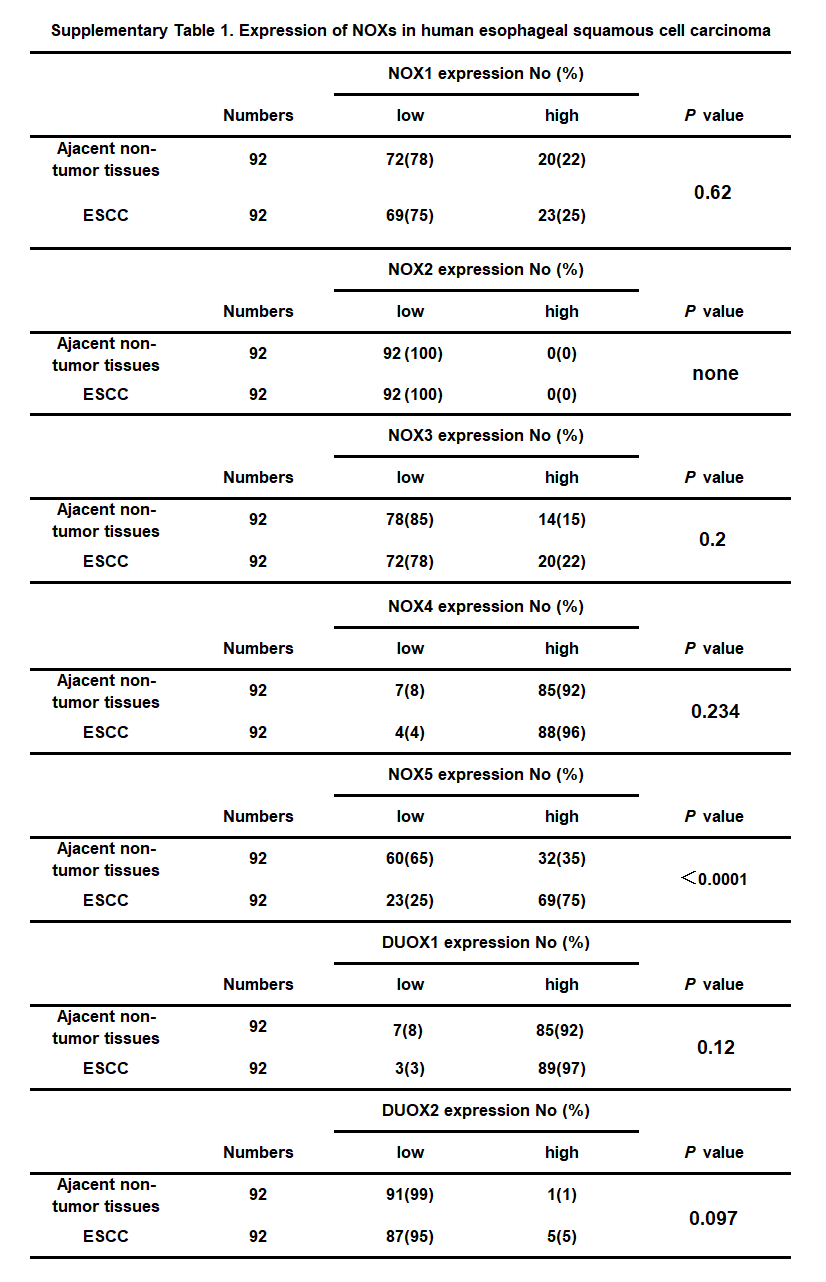

Supplement: Supplementary file 7 — supplementary Table 1 [file 41392_2020_193_MOESM7_ESM.tif]

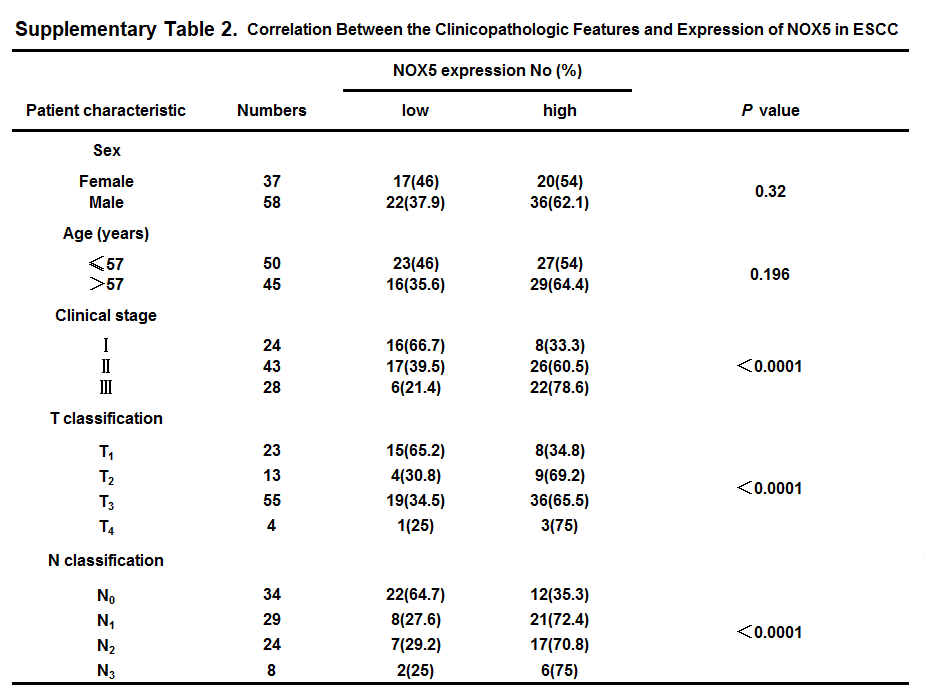

Supplement: Supplementary file 8 — supplementary Table 2 [file 41392_2020_193_MOESM8_ESM.tif]
